# Post-cholecystectomy major bile duct injury: ideal time to repair based on a multicentre randomized controlled trial with promising results

**DOI:** 10.1097/JS9.0000000000000403

**Published:** 2023-04-20

**Authors:** Mohammed A. Omar, Ayman Kamal, Alaa A. Redwan, Marwa N. Alansary, Emad Ali Ahmed

**Affiliations:** Departments of a General Surgery; b Anesthesia and Intensive Care, South Valley University, Qena; c Department of General Surgery, Helwan University, Helwan; d Department of General Surgery, Sohag University, Sohag, Egypt

**Keywords:** abdominal sepsis, bile duct injury, reconstruction time

## Abstract

Bile duct injury (BDI) is one of the serious complications of cholecystectomy procedures, which has a disastrous impact on long-term survival, health-related quality of life (QoL), healthcare costs as well as high rates of litigation. The standard treatment of major BDI is hepaticojejunostomy (HJ). Surgical outcomes depend on many factors, including the severity of the injury, the surgeons’ experiences, the patient’s condition, and the reconstruction time. The authors aimed to assess the impact of reconstruction time and abdominal sepsis control on the reconstruction success rate.

**Methods:**

This is a multicenter, multi-arm, parallel-group, randomized trial that included all consecutive patients treated with HJ for major post-cholecystectomy BDI from February 2014 to January 2022. Patients were randomized according to the time of reconstruction by HJ and abdominal sepsis control into group A (early reconstruction without sepsis control), group B (early reconstruction with sepsis control), and group C (delayed reconstruction). The primary outcome was successful reconstruction rate, while blood loss, HJ diameter, operative time, drainage amount, drain and stent duration, postoperative liver function tests, morbidity and mortality, number of admissions and interventions, hospital stay, total cost, and patient QoL were considered secondary outcomes.

**Results:**

Three hundred twenty one patients from three centres were randomized into three groups. Forty-four patients were excluded from the analysis, leaving 277 patients for intention to treat analysis. With univariate analysis, older age, male gender, laparoscopic cholecystectomy, conversion to open cholecystectomy, failure of intraoperative BDI recognition, Strasberg E4 classification, uncontrolled abdominal sepsis, secondary repair, end-to-side anastomosis, diameter of HJ (< 8 mm), non-stented anastomosis, and major complications were risk factors for successful reconstruction. With multivariate analysis, conversion to open cholecystectomy, uncontrolled sepsis, secondary repair, the small diameter of HJ, and non-stented anastomosis were the independent risk factors for the successful reconstruction. Also, group B patients showed decreased admission and intervention rates, decreased hospital stay, decreased total cost, and early improved patient QoL.

**Conclusion:**

Early reconstruction after abdominal sepsis control can be done safely at any time with comparable results for delayed reconstruction in addition to decreased total cost and improved patient QoL.

## Introduction

Highlights

- Early bile duct injury reconstruction after sepsis control can be done safely at any time.
- It has comparable results for delayed reconstruction.
- It has decreased re-intervention and admission, total hospital stays, and total cost.
- It results in improved patient quality of life early.

Bile duct injury (BDI) is one of the devastating complications of cholecystectomy procedures. It has a disastrous impact on long-term survival, health-related quality of life (QoL), healthcare costs as well as high rates of litigation^1^,^2^. Its incidence increased in laparoscopic cholecystectomy (LC), ranging from (0.3–1.5%), especially during the early phase of the surgeons’ training curve^3^–^5^. Based on the worldwide cholecystectomy frequency, even this low rate of BDIs presents a significant potential healthcare burden^6^.

The initial aim of BDI management is to control abdominal sepsis, followed by an assessment of BDI^7^. Strasberg *et al* 8. classified the BDIs into minor (Types A–D) and major injuries (Type E)^9^. Whereas minor injuries are usually treated with endoscopic or percutaneous intervention^10^, major injuries represent a significant challenge even for hepatobiliary surgeons (HBS)^6^,^11^, and reconstructive surgery by hepaticojejunostomy (HJ) is usually indicated^10^,^12^–^15^. Although the surgical outcomes are multifactorial-dependent, including the BDI type, referral time, patient’s condition, centres’ equipment, and surgeons’ experiences, reconstruction time is still the most important and debatable^1^,^2^,^11^,^13^,^15^–^21^. Many studies examined reconstruction time’s impact on short-term and long-term clinical outcomes, with varying and often contradictory findings^1^,^6^,^16^–^19^,^21^.

Theoretically, intraoperative reconstruction of fresh injuries allows the best surgical outcomes. In most cases, this is not the usual scenario and is challenging to achieve either because only a limited number of BDIs are diagnosed intraoperatively or because the working surgeon lacks the necessary experience to carry out such a repair^1^,^2^,^6^,^13^–^16^,^22^–^26^.

Additionally, most surgeons prefer delayed reconstruction for BDI diagnosed postoperatively, attributing this to a less inflamed surgical site with an adequate vascularized duct stump^1^,^2^,^19^,^24^,^27^. However, it may necessitate more interventions as preoperative optimization that leads to a more extended overall hospital stay and higher costs^6^,^28^. Once a BDI is diagnosed, both patient and surgeon feel anxious and fearful, and contrary to the desire of most surgeons, most patients insist that the injury be treated as quickly as possible with difficulty to convince them to wait, and they interpret any delay as a shift and an escape from the surgeon’s responsibility leading to high rates of litigation and extortion^11^.

In early reconstruction, the patient may be associated with sepsis and friable oedematous non-dilated common bile duct (CBD) stump, which is not practiced widely for fear of reconstruction failure^1^,^6^,^13^,^17^,^20^,^24^,^26^. Moreover, bile duct ischaemia might still develop at this time, which could later result in anastomotic stricture^18^. On the other hand, we cannot overlook its benefits in terms of a reduced burden for the patient and the surgeon, improving patients’ QOL, and avoiding the delayed reconstruction drawbacks^29^.

Unfortunately, the literature is extremely poor on specific guidelines for the reconstruction time of major BDI. Many papers recently reported a significant association between adequate preoperative control of abdominal sepsis and the surgical reconstruction success rate, with no effect of reconstruction time^1^,^30^.

In the absence of guidelines and patient medical knowledge and high rates of litigations and compensations, we believe that the decision for reconstruction time should be based on the predicted success, patient QoL, and the effective use of our low healthcare resources^6^,^22^. Therefore, we hypothesized that if abdominal sepsis is well-managed, BDI reconstruction can be done safely at any time. We aimed to present our experience in the surgical reconstruction of major post-cholecystectomy BDI and assess the impact of reconstruction time and abdominal sepsis control on the reconstruction success rate.

## Methods

This is a multicenter, multi-arm, parallel-group, randomized controlled trial (RCT). Ethics committee approval for the study was obtained from all centres. The study was registered in ClinicalTrials.gov (ID: NCT05436626). This work has been reported in line with the Consolidated Standards of Reporting Trials (CONSORT, Supplemental Digital Content 1, http://links.lww.com/JS9/A367, Supplemental Digital Content 2, http://links.lww.com/JS9/A368) reporting guideline^31^. Written informed consent was obtained from all participants.

### Trial design and participants

This study included all consecutive patients treated with an HJ for post-cholecystectomy BDI from February 2014 to January 2022 in 10 tertiary centres in Egypt. The inclusion criteria included patients diagnosed with type E1 to E4 BDI^8^ within six weeks after the cholecystectomy procedure, which failed to stent with endoscopic retrograde cholangiopancreatography. All participants were among the American Society of Anesthesiologists (ASA) scores I–III and agreed to complete the study. Patients with advanced liver cirrhosis, benign or malignant CBD stricture, or concomitant vascular or visceral injury were excluded. This study aims to evaluate the impact of HJ reconstruction time (early: within 6 weeks vs. delayed: after 6 weeks) of BDI and abdominal sepsis control (controlled vs. uncontrolled) on reconstruction success. Therefore, we defined three groups of patients including:

- Group A (Early reconstruction without sepsis control): all the included patients were operated on just after stabilization of the general condition (within 24 h).
- Group B (Early reconstruction with sepsis control): if the patients were presented with sepsis manifestations, expeditious sepsis control was achieved first, then reconstruction was performed. Sepsis control was obtained using broad-spectrum antibiotics, aggressive fluid resuscitation, and proper biliary and abdominal drainage. ICU admission with occasional pressors and transfusion might be indicated^10^.
- Group C (Delayed reconstruction): if the patients were presented with an abdominal collection, a preoperative ultrasound-guided tubal drainage was done to allow portal inflammation to subside until the planned reconstruction time. Re-laparoscopy or re-laparotomy for wash and drainage was required for patients with an excessive abdominal collection. Also, a biliary tree decompression by percutaneous transhepatic drain (PTD) was done in patients with cholangitis and/or long-standing cholestasis^10^,^24^.

### Data collection

Data collected included patient demographics, clinical presentations, initial cholecystectomy details, BDI criteria, operative details, postoperative course, and follow-up.

#### Preoperative assessment

BDI was suspected in patients who presented with post-cholecystectomy tachycardia, fever, abdominal pain, jaundice, bile leak, or abdominal bile collections^24^. All patients were subjected to thorough clinical, laboratory, and radiological [abdominal ultrasound and magnetic resonance cholangiopancreatography (MRCP)] evaluations to determine bile collections and the level of BDI^8^. Trans-tubal cholangiography was done for all patients with a biliary drain or a T-tube. A triphasic computed tomography scan was requested in a few cases with suspected vascular involvement. Occasionally, we performed an endoscopic retrograde cholangiopancreatography when we suspected that BDI might be managed non-operatively. We used the MDCalc website to diagnose abdominal sepsis and its severity (https://www.mdcalc.com/calc/1096/sirs-sepsis-septic-shock-criteria)^32^.

#### Operative techniques

The standard reconstruction technique was the Roux-en-Y HJ. It was performed as wide as possible, not under tension, and with good edge vascularity. It can be achieved by end-to-side or the Hepp–Couinaud approach. A division of the hilar plate and partial resection of segment IV were done when indicated to lengthen the bile duct stump and facilitate the biliary-enteric anastomosis^1^,^33^. In patients with a previously inserted PTD, this catheter was used as guidance for the damaged proximal end of the CBD and as a postoperative stent^11^. According to surgeon preference, an external trans-jejunal hepatic duct stent (6-10 French Nelaton catheter or naos-biliary catheter) was placed in some cases without prior PTD. The anastomosis was performed by a single layer of PDS (3/0 or 4/0) with continuous posterior and interrupted anterior sutures. Abdominal drains were placed in all patients. All reconstructions were performed by expert HBS with the same principle.

#### Postoperative management

Patients were transferred to the general surgical wards or the ICU according to their postoperative state. They received antibiotics intraoperatively and continued postoperatively for 10 days. Oral fluid intake was encouraged once intestinal sounds were audible. The daily follow-up plane include the following of the vital signs and drained outputs. We discharged the patients once oral intake was tolerated, liver function tests (LFTs) were normal, and the abdominal ultrasound revealed no abdominal collections. The external or the PTD catheter splint was removed once the cholangiogram revealed no bile leak or biliary stricture, maximally within three months^17^,^24^,^34^. We divided the complications into early (within 30 days after the definitive treatment) and late complications (after 30 days)^9^,^35^, and they were graded according to the Clavien–Dindo scoring system into minor (≤ grade II) and major complications (>Grade II)^36^.

#### Follow-up

Patients were followed-up during the first three months, biweekly, then monthly, during the first year, and then annually. The reconstruction was assessed especially for late-onset stricture by the clinical evaluation during each visit and laboratory, and radiologically (MRCP) when indicated.

#### Outcomes and manoeuvers

The primary outcome was a successful reconstruction rate. The secondary outcomes were blood loss, HJ diameter, operative time, drainage amount, drain and stent duration, postoperative LFTs, morbidity and mortality, number of admissions and interventions, hospital stay, patient QOL, and total cost.

The index treatment was our definitive intervention to repair the BDI^21^,^37^. It could be primary if the repair were the first without prior attempts, while secondary if one or more attempts were performed before the referral^1^,^11^,^38^.

For the assessment of the QoL, the Euro QoL-five dimensions (EQ-5D) score was used^39^,^40^. Repeated EQ-5D measurements were obtained at referral and on the 2nd, 4th, 6th, 8th, 10th, and 12th weeks. Patients were asked to select one statement that describes their health in each of the five dimensions. Based on the utility value of the Japanese and UK versions, which ranges from −0.111 (The worst) to 1.000 (full QOL), the answers were weighted and transformed to an EQ-5D score (details of conversion table are shown in appendix S1, Supplemental Digital Content 3, http://links.lww.com/JS9/A369)^39^,^41^.

According to the international study group of liver surgery, bile leakage is defined as fluid with an elevated bilirubin level (three times higher than the serum bilirubin measured at the same time) in the abdominal drain or the intra-abdominal fluid on or after postoperative day 3, or as the need for radiologic intervention because of biliary collections or re-laparotomy resulting from biliary peritonitis^16^. The anastomotic stricture was defined as radiological evidence of a stenosis at the HJ in MRCP or PTC in conjunction with abdominal pain, cholangitis, and abnormal LFTs, as well as the need for intervention in the form of dilatation, stenting, reoperation, or external drainage^1^,^21^,^35^. Cholangitis was defined according to the Tokyo guidelines^38^.

The successful reconstruction was defined as the repair with HJ with no further re-intervention up to the 3rd month postoperatively. In contrast, reconstruction failure was defined as any episodes of bile leak, cholangitis, jaundice, or liver abscess following the HJ that needed re-intervention within the first 3 months postoperatively^1^,^2^,^19^. Re-intervention was any radiological, endoscopic, or surgical intervention required after our repair during the follow-up study period^21^.

#### Sample size calculation

**Figure 1:**
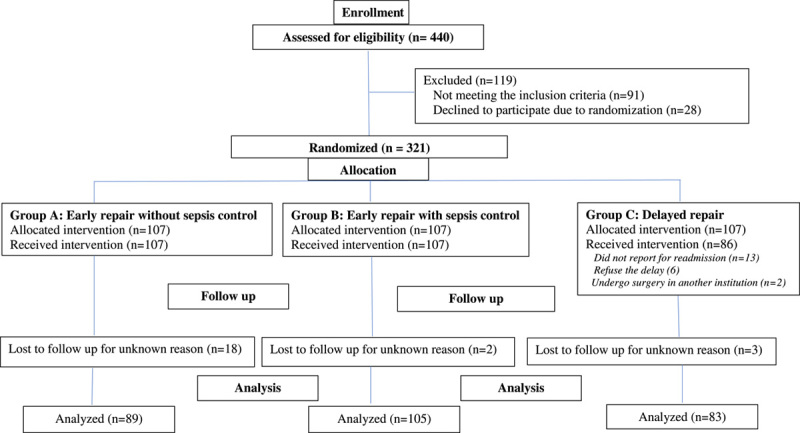
Flow of participants in the RCT According to CONSORT. RCT, randomized controlled trial.

We calculated the sample size with a power of 80% and a reliability of 0.05. We found that 81 patients should be included in each group. Eligible patients were randomly divided according to a computer-generated random number (Fig. 1)^2^,^19^. Allocation concealment was done with opaque, sealed envelopes by three junior doctors not involved in the eligibility and entry of patients.

### Statistical analysis

We used IBM SPSS statistics for Windows v. 25 (SPSS Inc.). We presented categorical data as numbers and percentages (%), and continuous data as either mean and SD for normally distributed data and median and interquartile range for non-normally distributed data. We used the one-way ANOVA, Kruskal–Wallis H test, and χ^2^ test as appropriate. Patient, initial cholecystectomy, BDI, operative, and postoperative data were factors analyzed to determine the risk factors for successful reconstruction. We analyzed the significant variables in the univariate analysis by a multivariate logistic regression model to detect the independent risk factors for successful reconstruction reporting as odds ratios (OR) with their 95% CI. The Kaplan–Meier was used for survival analysis to assess the time to re-intervention-free survivals, A *P* value less than or equal to 0.05 was considered statistically significant.

## Results

### Overall series

From 440 patients were evaluated for post-cholecystectomy BDI, 277 patients were included in the study and classified into three groups [89 patients (32.1%) in group A, 105 patients (37.9%) in group B, and 83 patients (30%) in the group C]. Our centres are tertiary referral centres in Egypt, which serve six governments with a population of about 25 million.

#### Preoperative data

Patient’s criteria, initial cholecystectomy criteria, and BDI criteria are summarized in Table 1. When the post-referral intervention was analyzed, patients in groups B and C underwent statistically significantly more interventions than those patients in group A; otherwise, there were no statistically significant differences between the three groups (Table 1).

**Table 1:** Patients demographics, initial cholecystectomy, and bile duct injury criteria.

Parameters	Group A (*n* = 89)	Group B (*n* = 105)	Group C (*n* = 83)	*P* value
Patients’ criteria				
Age (year)^a^	44.3 ± 4.9	43.2 ± 4.7	43.9 ± 5.9	0.34
Sex (female),^b^	58 (65.2)	64 (61)	52 (62.7)	0.81
BMI^a^	26.3 ± 2.1	26.8 ± 2.3	26.9 ± 2.4	0.79
ASA score^b^				0.6
ASA 1	55 (61.8)	72 (68.6)	54 (65.1)	
ASA II	29 (32.6)	29 (27.6)	25 (30.1)	
ASA III	5 (5.6)	4 (3.8)	4 (4.8)	
Clinical presentation^b^				
Bile leak	70 (78.7)	90 (85.7)	71 (85.5)	0.97
Abdominal collection	49 (55.1)	58 (55.2)	51 (61.4)	0.42
Obstructive jaundice	48 (53.9)	47 (44.8)	40 (48.2)	0.64
Abdominal pain	35 (39.3)	41 (39)	33 (39.8)	0.92
Others	19 (21.3)	10 (9.5)	14 (16.9)	0.13
Liver function test^a^				
TBIL (mg/dl)	6.34 ± 1.4	6.95 ± 1.6	6.56 ± 1.53	0.09
SGPT (IU/dl)	90.6 ± 24.2	92.02 ± 23.85	89.96 ± 23.77	0.56
SIRS manifestations^b^	61 (68.5)	65 (61.9)	52 (62.7)	0.56
Sepsis criteria	52 (58.4)	54 (51.4)	42 (50.6)	
Severe sepsis criteria	5 (5.6)	4 (3.8)	5 (6)	
Septic shock criteria	2 (2.2)	4 (3.8)	3 (3.6)	
Multiple organ dysfunction syndrome criteria	2 (2.2)	3 (2.9)	2 (2.4)	
EQ-5D score^c^	−0.111 (−0.111 to 0.483)	−0.111 (−0.111 to 0.483)	−0.111 (−0.111 to 0.483)	0.97
Initial cholecystectomy criteria				
Indication^b^				0.79
Chronic cholecystitis	68 (76.4)	84 (80)	63 (75.9)	
Acute cholecystitis	15 (16.9)	16 (15.2)	15 (18.1)	
Other	6 (6.7)	5 (4.8)	5 (6)	
Type^b^				0.58
Elective	70 (78.7)	88 (83.8)	67 (80.7)	
Emergency	19 (21.3)	17 (16.2)	16 (19.3)	
Procedure^b^				0.76
Laparoscopic	66 (74.2)	82 (78.1)	64 (77.1)	
Open	13 (14.6)	12 (11.4)	12 (14.5)	
Laparoscopic converted to open	10 (11.2)	11 (10.5)	7 (8.4)	
Place^b^				0.81
Our hospitals	3 (3.4)	2 (1.9)	2 (2.4)	
Other hospitals	86 (96.6)	103 (98.1)	81 (97.6)	
Primary surgeon^b^				0.37
Hepatobiliary surgeon	1 (1.1)	1 (1)	0 (0)	
General surgeon	88 (98.9)	104 (99)	83 (100)	
Intraoperative cholangiogram^b^	10 (11.2)	10 (9.5)	7 (8.4)	0.8
Bile duct injury criteria				
Time of diagnosis^b^				0.29
Intraoperative (pre-referral) diagnosis	12 (13.5)	14 (13.3)	7 (8.4)	
Post-perative (pre-referral) diagnosis	27 (30.3)	33 (31.4)	28 (33.7)	
Postoperative (post-referral) diagnosis	50 (56.2)	58 (65.2)	48 (57.8)	
Pre-referral intervention^b^				0.76
Patients	33 (37.1)	37 (35.2)	27 (32.5)	
Trial of repair	17	19	14	
Drainage	15	17	13	
Wash/drain - initial cholecystectomy	6	7	5	
Post-referral intervention^b^				
Patients	0	29 (27.6)	46 (55.4)	**0.001**
Drainage	0	29	46	
Re-laparotomy without repair	0	0	7	
Level of injury^b^				0.93
E1	15 (16.9)	17 (16.2)	13 (15.7)	
E2	70 (78.7)	83 (79)	66 (79.5)	
E3	1 (1.1)	3 (2.9)	3 (3.6)	
E4	3 (3.3)	2 (1.9)	1 (1.2)	
Time from injury to referral (day)^c^	8 (5–14)	8 (5–14)	8 (5–14)	0.12

Bold numerals indicate a statistically significant difference.

ASA, American Society of Anesthesiologists; EQ-5D, Euro Quality of life-5 dimentions score; SGPT, serum glutamic-pyruvic transaminase; SIRS, systemic inflammatory response syndrome; TBIL, total bilirubin.

a mean ± SD.

b *n* (%).

c median (Q1–Q3).

#### Operative data

There were statistically significant differences regarding all the operative parameters among the three groups except the type of index repair and SGPT level, which showed no statistically significant differences among the three groups (Table 2).

**Table 2:** Operative data.

Parameters	Group A (*n* = 89)	Group B (*n* = 105)	Group C (*n* = 83)	*P*_*1*_ value	*P*_*2*_ value	*P*_*3*_ value
Time from injury to reconstruction^a^	9 (5–15)	12 (10–18)	45 (43–46)	**0.03**	**0.001**	**0.001**
Type of index repair^b^				0.85	0.76	0.61
Primary repair	72 (80.9)	86 (81.9)	69 (83.1)			
Secondary repair	17 (19.1)	19 (18.1)	14 (16.9)			
Liver functions test^b^						
TBIL (mg/dl)	6.34 ± 1.4	4.95 ± 1.6	3.56 ± 1.2	**0.03**	**0.02**	0.07
SGPT (IU/dl)	90.6 ± 24.2	76.02 ± 19.85	62.96 ± 13.77	0.56	0.71	0.59
SIRS manifestations^c^	61 (68.5)	0 (0)	0 (0)	0.001	0.001	0.91
Sepsis criteria	52 (58.4)					
Severe sepsis criteria	5 (5.6)					
Septic shock criteria	2 (2.2)					
Multiple Organ Dysfunction Syndrome criteria	2 (2.2)					
Blood loss (ml)^b^	444.8 ± 117.9	320.9 ± 103.7	250.6 ± 87.8	**0.01**	**0.001**	**0.01**
The technique of anastomosis^c^				0.43	**0.007**	**0.009**
End-to-side	54 (60.7)	58 (55.2)	27 (32.5)			
Hepp–Couinaud	35 (39.3)	47 (44.8)	56 (67.5)			
Hepatic duct diameter (mm)^b^	7.9 ± 1.6	8.1 ± 2.2	8.6 ± 2.6	0.21	**0.01**	**0.03**
Hepaticojejunostomy diameter (mm)^b^	7.5 ± 2.2	7.9 ± 2	9.3 ± 2.9	0.67	**0.001**	**0.001**
Use of external stent^c^	72 (80.9)	91 (86.7)	46 (55.4)	0.68	**0.01**	**0.01**
PTD	6 (6.7)	14 (13.3)	32 (38.5)			
Trans-jejunal anastomotic drain	66 (74.2)	77 (73.4)	14 (16.9)			
No. trans-jejunal stents^c^				**0.01**	**0.001**	**0.01**
One	13 (14.6)	23 (21.9)	10 (12.1)			
Two	53 (59.6)	54 (51.4)	4 (4.8)			
Operative time (min)^b^	222.2 ± 59.3	193.6 ± 35.6	181.3 ± 57.3	**0.07**	**0.04**	0.67

Bold numerals indicate a statistically significant difference.

*P*_*1*_: represents the comparison between group A and B, *P*_*2*_: represents the comparison between group A and C, *P*_*3*_: represents the comparison between group B and C.

PTD, percutaneous transhepatic drainage; SGPT, serum glutamic-pyruvic transaminase; SIRS, systemic inflammatory response syndrome; TBIL, total bilirubin.

a median (Q1–Q3).

b mean ± SD.

c *n* (%).

#### Postoperative data

There were no statistically significant differences regarding the median time to resume oral intake on POD3 among the three groups, the mean time for stent removal, the mortality, and the follow-up duration. The median follow-up was two years, and the date of the last follow-up was recorded in April 2022. All the remaining postoperative parameters were statistically significantly different among the three groups (Table 3).

**Table 3:** Postoperative data.

Parameters	Group 1 (*n* = 89)	Group 2 (*n* = 105)	Group 3 (*n* = 83)	*P*_*1*_ value	*P*_*2*_ value	*P*_*3*_ value
Time to resume oral intake (day)^a^	3 (3–3)	3 (3–3)	3 (3–3)	0.71	0.72	0.76
Amount of drainage (ml)^a^	790 (520–1050)	270 (240–365)	200 (160–260)	**0.001**	**0.001**	0.09
Duration of the drain (day)^a^	11 (8–13)	5 (5–6)	4 (4–5)	**0.001**	**0.001**	0.91
Duration of the stent (day)^b^	69 ± 9.9	54.3 ± 7.8	49.1 ± 8.1	0.76	0.61	0.59
TBIL (mg/dl)^b^						
POD1	6.1 ± 1.4	4.05 ± 1.4	3.5 ± 1.2	**0.001**	**0.001**	0.08
POD5	3.5 ± 1	2.5 ± 0.9	2.1 ± 0.7	**0.001**	**0.001**	0.12
Postoperative complications^c^	67 (75.3)	45 (42.9)	34 (41)	**0.001**	**0.001**	0.81
Minor complications	27 (30.3)	18 (17.1)	9 (10.8)	**0.01**	**0.001**	0.61
Major complications	40 (44.9)	27 (25.7)	25 (30.1)	**0.01**	**0.01**	0.43
Mortality^c^	4 (4.5)	4 (3.8)	2 (2.4)	0.08	0.06	0.09
ICU admission (day)^a^	4 (4–5.5)	4 (1.5–4)	3.5 (1.5–3.6)	**0.01**	**0.001**	0.01
Hospital stays (day)^a^						
Index hospital stay	9 (7–11)	7 (6–9)	7 (6–9)	0.01	**0.01**	**0.89**
Total hospital stay	12 (9–16)	12 (10–14)	17 (11–26)	0.07	**0.01**	**0.01**
Total cost ($)^b^	4145 ± 714	3326 ± 683.8	4474 ± 822.9	0.001	0.67	0.001
EQ-5D^a^						
2nd week	0.052 (0.002–0.483)	0.052 (0.002–0.483)	−0.111 (−0.111 to 0.483)	0.67	**0.03**	**0.03**
4th week	0.730 (0.587–0.804)	0.804 (0.730–0.804)	−0.111 (−0.111 to 0.483)	**0.02**	**0.01**	**0.001**
6th week	0.804 (0.774–0.804)	0.804 (0.774–1)	−0.111 (−0.111 to 0.483)	**0.01**	**0.001**	**0.001**
8th week	0.804 (0.774–1)	0.804 (0.774–1)	0.052 (0.002–0.483)	0.87	**0.01**	**0.01**
10th week	0.804 (0.804–1)	1 (0.804–1)	0.804 (0.774–1)	0.63	**0.01**	**0.01**
12th week	0.804 (0.804–1)	1 (0.804–1)	1 (0.804–1)	0.54	0.56	0.79
Full QOL^a^						
6th week	15 (16.9)	36 (34)	0	**0.01**	**0.001**	**0.001**
8th week	29 (32.6)	43 (41)	0	0.09	**0.001**	**0.001**
10th week	38 (42.7)	67 (63.8)	28 (33.7)	**0.001**	0.09	**0.001**
12th week	55 (61.8)	81 (77.1)	55 (66.2)	**0.01**	0.32	**0.01**
Follow-up (month)^a^	26 (9-41)	23 (12-37)	25 (14-39)	0.67	0.81	0.78

Bold numerals indicate a statistically significant difference.

*P*_*1*_: represents the comparison between group A and B, *P*_*2*_: represents the comparison between group A and C, *P*_*3*_: represents the comparison between group B & C.

$, USD; POD1, Postoperative Day 1; POD5, Postoperative Day 5; TBIL, Total Bilirubin.

a Median (Q1–Q3).

b Mean ± SD.

c *n* (%).

#### Clavien–Dindo classification of postoperative complications

Clavien–Dindo grade 1 was the most common complication (42.7%) in large part to wound infection, followed by grade III (25.1%). There were statistically significantly higher grade I and grade III complication rates in group A when compared with groups B and C. At the same time, there were no differences in the remaining grades (II, IV, and V) between the three groups. Also, there was a statistically significant higher rate of complications per patient in group A compared with groups B and C (Table 4).

**Table 4:** Clavien–Dindo classification of postoperative complications.

			Group A (*n* = 89)		Group B (*n* = 105)		Group C (*n* = 83)		
Grades	Complications	*N* (%)	Early	Late	Early	Late	Early	Late	*P* value
Grade I	Biliary	114 (42.7)	26 (9.7)	0	14 (5.2)	1 (0.4)	11 (4.1)	0	**0.001**
	Bile leak grade A		10	0	5	0	3	0	
	Bile leak grade B		11	0	6	0	4	0	
	Cholangitis		3	0	3	0	4	0	
	Liver abscess		2	0	0	1	0	0	
	General		37 (13.9)	0	15 (5.6)	0	10 (3.7)	0	
	Wound infection		37	0	15	0	10	0	
Grade II	Biliary	61 (22.8)	10 (3.7)	5 (1.9)	8 (3)	3 (1.1)	4 (1.5)	3 (1.1)	0.61
	Cholangitis		2	1	2	2	1	1	
	Bile leak grade B		8	0	6	0	3	0	
	Intrahepatic stones		0	4	0	1	0	2	
	General		8 (3)	0	10 (3.7)	0	10 (3.7)	0	
	Chest infection		5	0	5	0	3	0	
	Paralytic ileus		2	0	3	0	3	0	
	DVT		0	0	0	0	1	0	
	UTI		1	0	2	0	3	0	
Grade III	Biliary	67 (25.1)	12 (4.5)	19 (7.1)	7 (2.6)	10 (3.7)	3 (1.1)	10 (3.7)	**0.01**
	Bile leak grade B (III a)		6	0	2	0	2	0	
	Bile leak grade C (III b)		6	0	3	0	1	0	
	Cholangitis (III a)		0	4	2	3	0	2	
	Anastomotic strictures (III b)		0	12	0	6	0	5	
	Liver abscess		0	3	0	1	0	3	
	General		0	0	0	2 (0.8)	1 (0.4)	3 (1.1)	
	Internal haemorrhage (III b)		0	0	0	0	1	0	
	Incisional hernia (III b)		0	0	0	2	0	2	
	Intestinal obstruction (III b)		0	0	0	0	0	1	
Grade IV	Biliary	15 (5.6)	1 (0.4)	0	0	1 (0.4)	0	1 (0.4)	0.73
	Liver cirrhosis (IV a)		1	0	0	1	0	1	
	General		4 (1.5)	0	3 (1.1)	0	5 (1.9)	0	
	ARDS (IV a)		3	0	2	0	4	0	
	Septic shock (IV b)		1	0	1	0	1	0	
Grade V	Biliary	10 (3.7)	2 (0.8)	0	1 (0.4)	1 (0.4)	0	1 (0.4)	0.81
	Septic shock		2	0	1	1	0	1	
	General		2 (0.8)	0	2 (0.8)	0	1 (0.4)	0	
	Sever arrhythmia		1	0	1	0	1	0	
	Pulmonary embolism		1	0	1	0	0	0	
Total		267 (100)	102 (38.2)	24 (9)	60 (22.5)	18 (6.7)	45 (16.9)	18 (6.7)	
Incidence of complications per patient		0.96	1.4		0.7		0.8		**0.01**

Bold numerals indicate a statistically significant difference.

ARDS, acute respiratory distress syndrome; DVT, deep venous thrombosis; UTI, urinary tract infection.

#### Postoperative success rate

The incidence of successful reconstruction was statistically significantly lower in group A (80.9%) compared with groups B (92.4%) and C (91.6%), which subsequently reflected in the incidence of re-intervention that was statistically significantly higher in group A (34.8%) compared with groups B (18.1%) and C (20.5%). No statistically significant differences existed between groups B and C. Anastomotic stricture and leak were the common causes of re-intervention in all groups. The number of unplanned readmissions and interventions was statistically significantly higher in group C compared with groups A and B, while there were no statistically significant differences between groups A and B (Table 5).

**Table 5:** Postoperative success, re-interventions, and admissions.

Parameters	Group A (*n* = 89)	Group B (*n* = 105)	Group C (*n* = 83)	*P*_1_ value	*P*_2_ value	*P*_3_ value
Successful reconstruction^a^	72 (80.9)	97 (92.4)	76 (91.6)	**0.01**	**0.01**	0.81
Re-intervention causes^a^	31 (34.8)	19 (18.1)	17 (20.5)	**0.001**	**0.001**	0.65
Anastomotic stricture	12 (13.5)	6 (5.7)	5 (6)			
Anastomotic leakage	12 (13.5)	5 (4.8)	3 (3.6)			
Cholangitis	4 (4.5)	5 (4.8)	2 (2.4)			
Liver abscess	3 (3.4)	1 (0.9)	3 (3.6)			
Incisional hernia	0 (0)	2 (1.9)	2 (2.4)			
Intestinal obstruction	0 (0)	0 (0)	1 (1.2)			
Internal haemorrhage	0 (0)	0 (0)	1 (1.2)			
No. unplanned readmissions per patient up to the index operation, *n*	1	1	2.4	0.9	**0.01**	**0.01**
No. unplanned readmissions up to the index operation^a^	89	105	201			
One admission	89 (100)	105 (100)	0 (0)			
> One admission	0 (0)	0 (0)	83 (100)			
No. interventions per patient up to the index operation, *n*	1	1.3	1.6	0.08	**0.01**	**0.01**
No. interventions up to the index operation^a^	89	134	136			
One procedure	89 (100)	76 (72.4)	37 (44.6)			
> One procedure	0 (0)	29 (27.6)	46 (55.4)			
Total number of unplanned readmissions admissions per patient, *n*	1.4	1.5	2.6	0.73	**0.01**	**0.01**
Total number of unplanned readmissions (last follow up)^a^	121	153	218			
One admission	58 (65.2)	64 (61)	0 (0)			
> One admission	31 (34.8)	41 (39)	83 (100)			
Total number of interventions per patient, *n*	1.4	1.5	1.8	0.32	**0.01**	**0.01**
Total number of interventions (last follow up)^a^	121	153	153			
One procedure	58 (65.2)	64 (61)	35 (42.2)			
> One procedure	31 (34.8)	41 (39)	48 (57.8)			

Bold numerals indicate a statistically significant difference.

*P*_*1*_: represents the comparison between group A and B, *P*_*2*_: represents the comparison between group A and C, *P*_*3*_: represents the comparison between group B and C.

a *n* (%).

**Figure 2:**
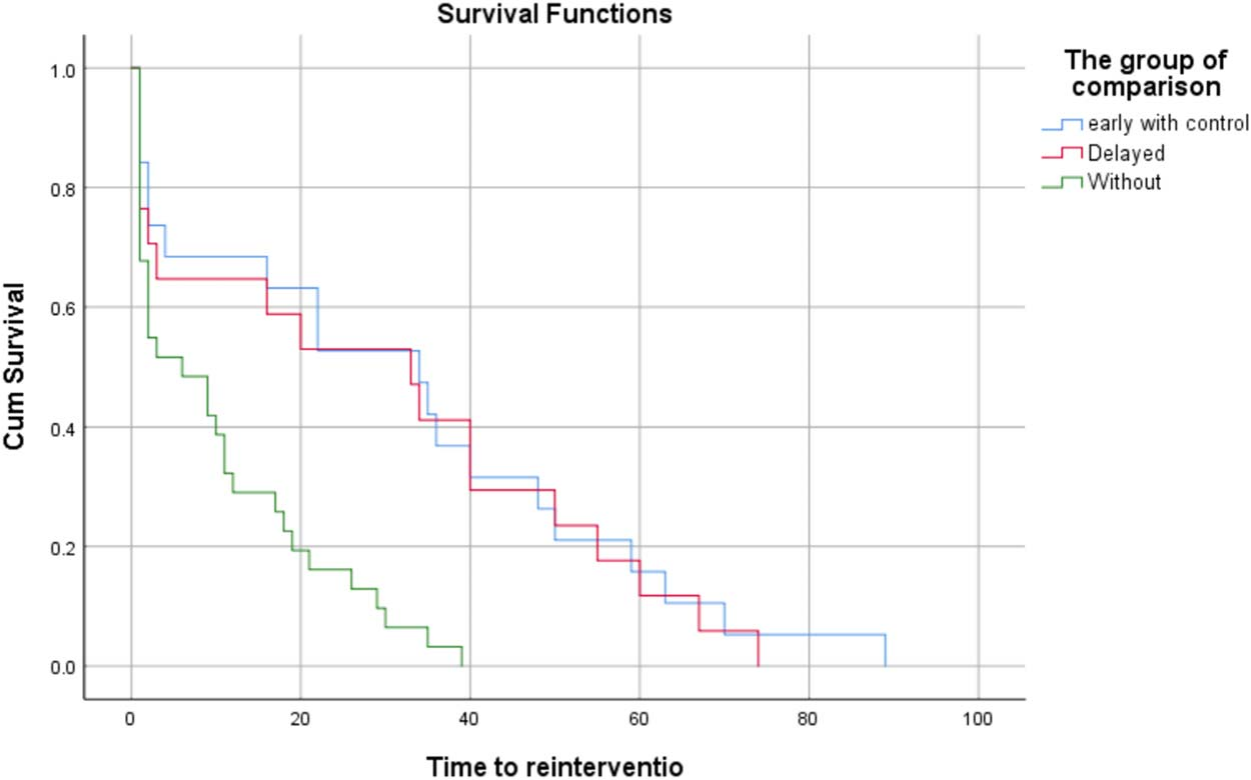
Time to re-intervention survival analysis.

Patients who underwent early reconstruction with sepsis control had a median time to re-intervention of 34 (95% CI, 13.7–54.3) months. This was nearly equal to patients who underwent delayed reconstruction 33 (95% CI, 8.8–57.2) months. Both groups have a median time to re-intervention longer than patients who underwent early reconstruction without sepsis control 6 (95% CI, 0.5–11.4) months. The survival distributions for the three groups were statistically significantly different X^2^(2) = 18.27, *P* less than 0.001 (Fig. 2).

#### Univariate and multivariate analysis for successful reconstruction

Study variables associated with successful reconstruction at the *P* = 0.25 univariate level of statistical significance were included in a multivariate logistic regression model. With univariate analysis, older age, male gender, LC, conversion to open cholecystectomy, failure of intraoperative BDI recognition, Strasberg E4 classification, uncontrolled abdominal sepsis, secondary repair, end-to-side anastomosis, the diameter of HJ (<8 mm), non-stented anastomosis, and major complications were risk factors for successful reconstruction. With multivariate analysis, conversion to open cholecystectomy, uncontrolled sepsis, secondary repair, small diameter of HJ, and non-stented anastomosis were the independent risk factors for the successful reconstruction (Table 6).

**Table 6:** Univariate and multivariate analysis for risk factors for successful reconstruction.

	Univariable analysis		Multivariable analysis	
Independent variables	OR (95% CI)	*P* value	OR (95% CI)	*P* value
Age, < 45 vs. > 45 years	0.87 (0.76–1.23)	**0.007**	1.21 (1.02–1.44)	0.09
Sex, female vs. male	0.96 (0.43–2.1)	**0.01**	0.83 (0.35–2.01)	0.69
BMI, < 25 vs. > 25	1.26 (0.97–1.67)	0.75	1.11 (0.92–1.34)	0.27
ASA score				
ASA I vs. ASA II	0.86 (0.34–4.35)	0.37	1.99 (0.06–6.07)	0.69
ASA I vs. ASA III	1.21 (0.96–6.37)	0.19	1.80 (0.98–7.85)	0.08
Type of cholecystectomy, elective vs. emergency	1.67 (1.19–2.54)	0.31	1.71 (1.22–2.71)	0.33
Indication of cholecystectomy				
Chronic vs. acute cholecystitis	1.39 (0.93–2.17)	0.91	1.03 (0.62–1.61)	0.96
Chronic vs. other	1.17 (0.91–1.65)	0.46	1.27 (0.83–2.01)	0.37
Initial cholecystectomy				
Open vs. laparoscopic	0.96 (0.45–3.31)	**0.02**	1.67 (0.32–5.35)	0.09
Laparoscopic converted to open, yes vs. no	1.41 (0.61–6. 71)	**0.01**	1.87 (0.96–5.37)	**0.01**
Intraoperative BDI recognition, yes vs. no	0.78 (0.33–1.22)	**0.02**	0.89 (0.45–2.25)	0.51
Level of BDI				
E1 vs. E2	0.23 (0.17–0.98)	0.32	0.24 (0.04–1.41)	0.11
E1 vs. E3	0.32 (0.13–1.19)	0.09	0.39 (0.08–1.89)	0.67
E1 vs. E4	0.98 (0.77–1.78)	**0.02**	1.34 (1.03–1.98)	0.14
Reconstruction time, delayed vs. early	1.19 (0.54–2.57)	0.65	0.55 (0.19–1.57)	0.26
Abdominal sepsis, yes vs. no	2.54 (0.9–7.16)	**0.01**	2.98 (1.13–9.76)	**0.001**
Type of index repair, primary vs. secondary repair	1.91 (0.47–7.64)	**0.03**	3.69 (0.72–8.86)	**0.01**
Estimated blood loss, ≤ 250 vs. > 250 ml	0.31 (0.12–1.23)	0.09	0.39 (0.09–2.06)	0.23
The technique of anastomosis, end-to-side vs. Hepp–Couinaud	1.42 (0.39–5.13)	**0.01**	5.84 (0.29–6.65)	0.07
Diameter of HJ, > 8 vs. < 8 mm	1.02 (0.34–2.19)	**0.04**	0.64 (0.41–5.02)	**0.02**
Use of external stent				
Yes vs. no	1.8 (0.51–6.34)	**0.01**	2.3 (1.22–8.97)	**0.01**
PTD vs. trans-jejunal stent	1.80 (0.82–3.93)	0.13	2.96 (0.35–4.67)	0.31
Number of stents, one vs. two	0.91 (0.56–1.79)	0.21	0.98 (0.31–3.23)	0.98
Operative time	2.36 (1.67–5.19)	0.51	0.986 (0.96–1.01)	0.09
Grade of complications, minor vs. major	7.8 (2.65–25.05)	**0.001**	7.1 (2.05–24.29)	0.12

Bold number: indicate the significant result.

ASA, American Society of Anesthesiologists; BDI, bile duct injuty; HJ, hepaticojejunostomy; PTD, percutaneous transhepatic drainage.

## Discussion

The incidence of BDI has significantly increased internationally during the past 15 years^11^. In Egypt, LC is developing rapidly in primary hospitals with under-equipped medical infrastructure and a low learning curve for practicing surgeons. As a result, there was an increase in the BDI frequency despite our attempts to spread the practice of safe LC. Furthermore, the majority were significant and complex injuries. Therefore, these patients must promptly recognize their problem and find reliable treatment with a long-term success rate^3^–^5^. The safe and efficient BDI reconstruction is a continuing topic of conflict. No published guidelines have revealed relevant factors to ensure an optimal outcome. With a particular emphasis on reconstruction time, many published studies examined the impact of many factors on the success rate of BDI reconstruction^1^,^6^,^16^,^17^,^42^. However, despite its significance, the impact of abdominal sepsis control was ignored or briefly discussed.

El Nakeeb *et al* 17. Studied 412 patients who underwent HJ for BDI and found that favourable results were more frequently seen in the immediate and delayed reconstruction. A recent systematic review and meta-analysis reported early, and delayed reconstruction is associated with lower postoperative morbidity and anastomotic stricture rates than intermediate reconstruction^18^. Dominguez-Rosado *et al* 1. revealed that the incidence of postoperative morbidities before the primary reconstruction was reduced by managing sepsis and avoiding biliary stents. According to their findings, intermediate biliary reconstruction should be avoided to decrease postoperative morbidities. Also, de Reuver *et al* 11. and Felekouras *et al* 20. have shown that patients with early or delayed reconstruction can achieve the same outcomes. Battal *et al* 26. and Cho *et al* 42. recommended early reconstruction when conditions permit, while Iannelli *et al* 2. showed that optimum outcomes can be achieved with delayed reconstruction.

On the contrary, A large retrospective multicentre study^16^ showed that the reconstruction time did not impact postoperative morbidity, mortality, anastomotic patency, or re-intervention rate. Stewart and Way^30^ concluded that in the presence of abdominal sepsis control, cholangiography, good surgical technique, and an experienced HBS, BDI reconstruction could be performed safely at any time and there was no excuse for delaying the reconstruction. Sicklick *et al* 23. concluded that the timing of the operation, presenting symptoms, and prior repair history had no impact on the incidence of postoperative complications or length of hospital stay.

Many papers revealed that abdominal sepsis control is a critical preventive measure for postoperative complications and reconstruction failure^1^,^30^,^34^. Sulpice *et al* 9. revealed that abdominal sepsis and liver cirrhosis were independent risk factors for significant morbidities and anastomotic stricture, respectively. Thomson *et al* 29. reported no difference in results between patients who underwent surgery within 2 weeks and those who waited until sepsis and peritoneal soiling subsided when a professional HBS performed the repair procedure.

The literature has a highly variable cutoff time for BDI reconstruction. For early repair, the reported time is between 2 and 45 days or less^1^,^2^,^6^,^13^,^15^,^23^; for intermediate repair, it is between 8 days and 6 weeks^1^; and for delayed repair, it is more than 2 days to more than 6 weeks^1^,^2^,^6^,^13^,^15^,^23^. Without a universal definition, it is challenging to compare one study to another directly^35^. In our research, based on earlier articles^1^,^2^,^11^,^19^, we adopted two-time frames for early (less than 6 weeks) and delayed (more than 6 weeks) reconstruction based on the hypothesis that this will be sufficient time for optimal reconstruction^2^,^11^.

### Patient demographics

This study revealed a higher incidence of BDI in females (62.8%), which may be attributed to the high incidence of gallstones and cholecystectomies in females, consistent with many papers 1,10,16,17,21,34. The incidence of BDI was more in chronic cholecystitis (77.6%) and elective cholecystectomies (81.2%), and this may be attributed to the primary and secondary hospitals (97.7%), which still adopt the conservative treatment of acute cholecystitis, and this was consistent with many papers reported that chronic cholecystitis was the most frequent indication for initial cholecystectomy^1^,^17^,^35^. Recently it was reported that LC is associated with increased incidence and complexity of BDI^1^,^2^,^10^,^12^,^21^, and this was consistent with our results that reported 86.6% of our cases were after LC. This may be explained based on the referral hospitals with general surgeons (99.5%) with an early learning curve of laparoscopy that reduces the ability to recognize landmarks and successfully carry out the procedure. On the contrary, many studies reported that BDI was higher after open cholecystectomy, and they explained their results based on the restriction of open cholecystectomy for complex and challenging cases^1^,^17^,^43^. The estimated frequency of intraoperative BDI detection varies from 7 to 90%, which is attributed to the difference between the centres performing intraoperative cholangiogram (IOC) as a routine^1^,^3^,^4^,^10^. In our study, the incidence of intraoperative cholangiogram was very low (9.7%), which may explain the low incidence of intraoperative BDI diagnosis (11.9%). Fifty patients (31%) had a local attempt to correct the BDI, but leakage continued, and they were referred to our centres for secondary repair. Radiographic mapping of the biliary tree was obtained in every patient after being sent to our hospitals, and Strasburg level E2 injury was the most frequent (79.1%). The median referral time was 8 days (interquartile range 5–14 days), consistent with many previous studies^11^,^16^,^34^.

### Operative data

Surgical reconstruction is the most effective treatment of major BDI with optimal long-term outcomes^9^,^44^. In our study, both end-to-side and Hepp–Couinaud techniques for HJ were used equally. However, the end-to-side HJ was the procedure of choice in groups A and B, while the Hepp–Couinaud technique was the procedure of choice in group C. Myburgh reported that the Hepp–Couinaud technique is the procedure of choice^45^. Also, Mercado *et al* 46 recommended the Hepp–Couinaud technique, as early biliary repair of non-dilated bile ducts is technically challenging and difficult to conduct below the confluence with more failure rate compared with HJ at or above the confluence.

Contrary to popular belief, some studies reported that dilated bile duct did not prevent stricture after HJ^47^,^48^. Studies reported^19^,^49^ that diagnosing a dilated bile duct could not be made with accuracy because it should be titrated in response to the average baseline size, various imaging techniques, post-cholecystectomy normal dilation, and interobserver variability. Most published studies did not record the bile duct diameter^19^. This study used a Nelaton plastic catheter and a balloon biliary dilator intraoperatively to assess the bile duct’s actual diameter and obtain the maximum dilatation for the subsequent reconstruction. The bile duct and HJ diameter were statistically significantly narrower in groups A and B.

The use of trans-anastomotic stents is still debatable^34^,^50^–^52^. Some studies^34^,^53^–^55^ recommended the routine use of a trans-anastomotic stent. They reported that stents allow adequate biliary drainage and lower the biliary pressure, which protects the integrity of the anastomosis, allows easy access for subsequent interventions, and improve short and long-term outcomes. Other studies^1^,^51^,^56^,^57^ reported equivalent outcomes without stenting. It is impossible to determine if this results from the technically complex repair, in which the surgeon elected to insert the stent as a preventive measure, or if it is a cause. In this study, we used trans-anastomotic external jejunal stents in 157 patients (56.7%), and PTD in 52 patients (18.8%) based on surgeon preference and the diameter of intrahepatic channels.

### Postoperative morbidity and mortality

Published studies reported 1.5–3 months for stent removal after doing a cholangiogram and ensuring anatomical and functional patency of the anastomosis^17^,^34^. This was consistent with our results, although group A was longer (69±9.9 vs. 54.3±7.8 and 49.1±8.1), but without statistically significant difference.

The reported rate of postoperative morbidity after BDI reconstruction ranged from 15% to 65%^18^,^23^,^37^. Many recent studies^2^,^4^,^16^,^18^,^58^ reported a statistically significant lower morbidity rate in delayed reconstruction. On the other hand, a recent meta-analysis and systematic review^19^ reported no significant difference in the rate of morbidity between early and delayed reconstruction (22.7% vs. 16.3%; OR 1.34, 95% CI 0.9–1.8, *P*=0.08). Also, Dominguez-Rosado *et al* 1. reported that intermediate reconstruction is not a risk factor for complications compared with early and delayed reconstruction. Booij *et al* 35. documented that the reconstruction time did not influence short and long-term outcomes after HJ. In this study, postoperative complications affected 52.7% of patients. Group A patients were associated with a statistically higher risk of morbidity (75.3%) compared with groups B and C (42.9% and 41%). At the same time, there was no statistically significant difference between groups B and C.

Dominguez-Rosado *et al* 1. reported that wound infection was the most frequent (16%), followed by pneumonia (5%). In this study, wound infection was the most frequent general complication (22.4%). A recent meta-analysis and systematic review^19^ revealed that patients who repaired early had a statistically significantly higher incidence of bile leak than those with delayed repair. El Nakeeb *et al* 17. reported that anastomotic leakage was significantly more in intermediate BDI reconstruction. On the other hand, many studies^15^,^16^,^35^ reported that leakage did not differ considerably between early, intermediate, or delayed repairs. Our study revealed a statistically significant higher bile leak in group A patients compared with groups B and C. A recent meta-analysis^18^. reported that intermediate reconstruction was associated with a higher risk of stricture than delayed reconstruction. Also, many recent studies^17^,^27^,^35^,^59^ reported statistically significantly lower stricture rates in delayed reconstruction. On the other hand, A recent meta-analysis and systematic review^19^ found no statistically significant association between an early repair and a stricture incidence that was approximately twice as high as that associated with the delayed repair, while Perera *et al* 15. reported statistically significantly lower rates of stricture in patients having reconstruction between 1 and 3 weeks. In this study, the reported stricture rate was 8.3% of patients, and this result was in line with previously published studies that reported stricture rates varying between 5 and 70%^9^,^14^,^37^,^43^,^54^. Our study revealed a statistically significant higher stricture rate in group A patients (13.5%) compared with group B (5.7%) and group C (6%) patients.

A recent meta-analysis^18^. reported a mortality rate ranging from 0 to 18%, and this was in line with our study, which revealed a mortality rate of 3.6% of patients. Two recent studies^17^,^18^ reported no significant relationship between the timing of BDI reconstruction and mortality rate. On the other hand, Ismael *et al* 25. found a statistically significantly lower mortality rate in patients undergoing delayed reconstruction. This study showed no significant association between the reconstruction time and the mortality rate.

Degeforde *et al* 22. reported shorter hospitalization in early reconstruction. This study agreed with this result and revealed shorter hospital stay for group B patients, followed by group A patients, and lastly group C patients. Group C patients in whom we intend to post-pone reconstruction are closely monitored to ensure drain patency and good nutritional condition. Many patients with social and financial problems stay in the hospital until the repair is complete.

Even though the cost burden is a growing concern^22^,^60^, its relationships to the reconstruction time have received little attention and discussion^19^. Degeforde *et al* 22. proved the cost-effectiveness of early repair by HBS. Similarly, this study confirmed the reduced total cost of group B patients. The driving force for this reduction was the ability to return the patient to a relatively normal life with as few interventions and hospital admission as possible. Furthermore, the cost of group A and C patients was influenced by the increased number of interventions and hospital admissions.

The long-term impact of BDI on health-related QoL has been studied in several papers^14^. Although some authors^28^,^61^ reported no effect of BDI on the QoL, the majority demonstrate a poorer QoL, even years after the BDI has been treated^62^–^66^. Additionally, Booij *et al* 60. reported work-related restrictions, a loss of productivity in work, and an increase in the utilization of disability benefits after BDI. One of the several factors that affect the QoL is the reconstruction time. Rystedt and Montgomery^67^ showed that the early reconstruction resulted in QoL comparable to patients who underwent uncomplicated cholecystectomy. Our study revealed that the QoL in group B patients was significantly improved on all five parameters of the EQ-5D scale from the 2nd week, and only comparable results in all groups were achieved in the 12th week. Also, the full QoL was achieved significantly early in group B patients.

Dominguez-Rosado *et al* 1. reported that failed reconstruction occurred in 132 patients (21.5%). A recent meta-analysis and systematic review^19^ reported that the rate of reconstruction failure with early reconstruction was significantly higher than that with delayed reconstruction, and this rate of reconstruction failure did not differ significantly between groups when data analyses were restricted to a homogeneous population who underwent repair by HBS. This study revealed a successful reconstruction rate of 88.4% of patients. There was a statistically significant lower reconstruction success rate in group A than in groups B and C. At the same time, there was no statistically significant difference between groups B and C.

Many papers^4^,^16^,^35^,^42^ reported that a re-intervention rate was required in 7–31% of patients, and the median time up to the first reconstruction failure was 4–22 months. They showed that the timing of reconstruction did not affect the need for re-intervention. On the contrary, A recent meta-analysis and systematic review^19^ reported that more patients in the early-repair group required more re-interventions compared with those in the delayed-repair group. Fischer *et al* 10. reported that patients in the delayed group underwent substantially more interventions than patients in the early group when the total number of interventions carried out before referral was analyzed. Our result revealed a re-intervention rate of 24.2%. there were a statistically significant higher re-intervention rate and shorter median survival for re-intervention in group A, and comparable result in groups B and C. Anastomotic stricture and leakage were the commonest cause for re-intervention. Also, when the number of interventions up to the index operation and total up to the last follow-up was analyzed, patients in group C underwent significantly more interventions compared to groups A and B. El Nakeeb *et al* 17. reported that re-admission was statistically significantly frequent in intermediate BDI reconstruction. In this study, when the number of unplanned readmissions up to the index operation and total up to the last follow-up was analyzed, patients in group C underwent significantly more admission compared with groups A and B.

### Univariate and multivariate analysis

Two studies^1^,^68^ reported that conversion to open surgery was associated with reconstruction failure and more complex BDI. Similarly, our study revealed that conversion to open surgery was a significant risk factor for successful reconstruction in multivariate analysis (OR 1.87, 95 CI 0.96–5.37, *P* < 0.01).

Many previous studies^11^,^12^,^16^,^17^,^25^,^30^,^35^,^69^ showed a significant association between poor reconstructive surgery outcomes and proximal BDI. Sultan *et al* 70. reported a higher failure rate of HJ for E3 and E4 BDI but not statistically significant. Our study revealed no association between the level of BDI and successful reconstruction. Successful surgical reconstruction of Type E injuries can be as high as 90% when performed in highly specialized centres with expert HBS^17^,^71^. Unfortunately, many BDIs are managed by injuring nonspecialized surgeons in nonspecialized centres with poor surgical outcomes^10^,^23^,^72^,^73^.

The optimal time for BDI reconstruction remains controversial. Some authors advise delayed repair^11^,^74^, some authors suggested early repair^15^,^26^,^35^,^42^, and others reported no difference^6^,^15^,^23^,^30^,^34^,^75^–^77^. The different results on the impact of reconstruction time could be attributed to the differences in the definitions of the reconstruction time, morbidities, and anastomotic failure, as well as small sample sizes and different study designs^13^,^20^,^29^,^30^. This study agreed with the previous results that revealed no impact of reconstruction time on the success rate (OR 0.55, 95% CI 0.19–1.57, *P* = 0.26).

Many papers^19^,^34^,^77^ revealed that uncontrolled bile leak or sepsis could make anastomosis extremely difficult to be done or heal for all surgeons regardless of their expertise. Furthermore, even with aggressive management, it may still be active following surgery, predisposing patients to fibrosis and late anastomotic stricture^19^,^34^. On the contrary, several studies^1^,^30^,^35^,^78^ reported that complete eradication of abdominal sepsis was protective against reconstruction failure irrespective of the timing of surgical reconstruction. This study agreed with many papers^1^,^9^,^30^,^75^,^77^ and concluded that abdominal sepsis is a significant predictor for successful reconstruction (OR 2.98, 95% CI 1.13–9.76, *P* = 0.001).

Two studies^1^,^9^ reported no impact of the pre-referral intervention on the outcome. On the contrary, many studies^10^,^11^,^79^,^80^ reported an increased incidence of major postoperative complications and HJ failure in patients with prior repair attempts. This study revealed secondary repair as an independent risk factor for successful reconstruction (OR 3.69, 95% CI 0.72–8.86, *P* = 0.01).

Gad *et al* 34. revealed a significant association between operative bleeding and successful reconstruction. They explained this association based on the high incidence of hepatectomy and liver cirrhosis in this case. Our study revealed no association between intraoperative bleeding and successful reconstruction.

Two recent studies^70^,^81^ reported that the Hepp–Couinaud technique is superior to end-to-side anastomosis and is associated with good results on long-term outcomes. The wider stoma, tension-free anastomosis, and good vascularity may explain this^14^. Our study revealed that the Hepp–Couinaud technique is better.

Two recent studies^1^,^51^ reported a better outcome of Roux-en-Y HJ without using stents. On the contrary, many papers^34^,^53^–^55^ reported that stenting was associated with a better outcome. Our study demonstrated trans-anastomotic stent as an independent predictor for successful reconstruction (OR 2.3, 95% CI 1.22–8.97, *P* < 0.01). de Reuver *et al* 82. revealed better outcomes after more than one anastomotic stent insertion. On the contrary, this study showed no impact of the number of anastomotic stents on the success rate.

Several studies^15^,^20^,^23^,^30^,^59^,^83^ reported that the BDI reconstruction by an experienced HPB surgeon in a specialized centre is associated with a significantly better surgical outcome. They explained this better outcome based on the availability of a multidisciplinary team, including the HPB surgeon, gastroenterologist, endoscopist, and interventional radiologist^17^. Also, the HPB surgeons’ experiences and their surgical HJ reconstruction technique are superior to the end-to-end anastomosis technique, which most general surgeons prefer. Along the same line, our promising results for early repair may be attributed to surgical reconstructions that an experienced HBS did in well-equipped tertiary centres.

### Strengths and limitations of the study

Our study has several strengths. To the best of our knowledge, this is one of the early RCTs evaluating the impact of reconstruction time and abdominal sepsis control on reconstruction success. It exclusively considers HJ reconstruction and excludes other reconstruction methods, creating a homogenous study. We investigated many short-term and long-term complications. Finally, our collected data from our collaborating centres had the exact homogeneous timing and endpoint. These circumstances make our study unique and raise the likelihood that conclusions will be valid. On the contrary, our study has a few limitations that should also be acknowledged. The first was the relatively short duration of the follow-up. Therefore, the outcomes examined were those that occur in the follow-up period and long-term complications such as biliary stricture, intrahepatic stones, biliary cirrhosis, and liver cell failure need a long follow-up period (up to 10 years) to be assessed. Secondly, there was no adjustment for possible confounders such as vascular injury. Thirdly, the low incidences of some complications may compromise the statistical results. Fourthly, there is no standardized procedure for reporting the outcomes for better comparison with other studies. Finally, The use of trans-anastomotic external jejunal stents and PTD based on surgeon preference and diameter of intrahepatic duct is a potential bias.

## Conclusion

In conclusion, the results of this multicenter RCT revealed that avoidance of conversion to open surgery, controlled abdominal sepsis, avoidance of pre-referral repair attempts, wide anastomotic stoma, trans-anastomotic stent, and repair by a skilled HBS in tertiary centres are the most crucial factors associated with successful reconstruction. Once the variables mentioned above had been reached, the timing of the repair did not affect the reconstruction’s success. Early reconstruction after sepsis control can be done safely at any time with comparable results for delayed reconstruction in addition to decreased re-intervention and admission, total hospital stays, total cost, and improved patient QoL early.

## Ethical approval

Ethical committee approval (Faculty of Medicine—South Valley University) for the study was obtained with this identification number: (SVU/MED/SUR011/4/489). The study was registered in the ClinicalTrials.gov database with this identification number: NCT05436626 https://clinicaltrials.gov/ct2/show/NCT05436626

## Source of funding

This research did not receive any specific grant from funding agencies in the public, commercial, or not-for-profit sectors.

## Author contribution

Study concept and design: All authors. Data collection: All authors. Analysis and interpretation of data: M.A. O. Writing and literature review: M.A.O. Review and corrections: M.A. O., A.A. R., E.A.A. Final approval of the version: A.A.R.

## Conflicts of interest disclosure

We certify that there is no conflict of interest with any financial/ research/academic organization, with regards to the content/research work discussed in the manuscript.

## Guarantor

Dr. Mohammed Ahmed Omar.

## Data statement

All data statement are available when requested.

## Provenance and peer review

Not commissioned, externally peer-reviewed.

## Supplementary Material
