# Supplementary material for: Post-cholecystectomy major bile duct injury: ideal time to repair based on a multicentre randomized controlled trial with promising results
Source: Int J Surg. 2023 Apr 20;109(5):1208–21. doi: 10.1097/JS9.0000000000000403 (PMC10389623; doi:10.1097/JS9.0000000000000403)
Supplement: Supplementary file 1 [file js9-109-1208-s001.docx]

**Enrollment**

**Assessed for eligibility (n= 440)**

Excluded (n=119)

Not meeting the inclusion criteria (n=91)

Declined to participate due to randomization (n=28)

**Randomized (n = 321)**

**Allocation**

**Group C: Delayed repair**

Allocated intervention (n=107)

Received intervention (n=86)

*Did not report for readmission (n=13)*

*Refuse the delay (6)*

*Undergo surgery in another institution (n=2)*

**Group B: Early repair with sepsis control**

Allocated intervention (n=107)

Received intervention (n=107)

**Group A: Early repair without sepsis control**

Allocated intervention (n=107)

Received intervention (n=107)

**Follow up**

**Follow up**

Lost to follow up for unknown reason (n=3)

Lost to follow up for unknown reason (n=2)

Lost to follow up for unknown reason (n=18)

**Analysis**

**Analysis**

Analyzed (n=105)

Analyzed (n=89)

Analyzed (n=83)

**Fig 1:** Flow of participants in the RCT According to CONSORT.
