# Supplementary material for: Post-cholecystectomy major bile duct injury: ideal time to repair based on a multicentre randomized controlled trial with promising results
Source: Int J Surg. 2023 Apr 20;109(5):1208–21. doi: 10.1097/JS9.0000000000000403 (PMC10389623; doi:10.1097/JS9.0000000000000403)
Supplement: Supplementary file 3 [file js9-109-1208-s003.docx]

**Appendix 1**

A conversion table of EQ-5D score in Japan

| **5Dimen-tions** | **Utility value** | **5Dimen-tions** | **Utility value** | | **5Dimen-tions** | **Utility**  **value** | **5Dimen-tions** | **Utility**  **value** | **5Dimen-tions** | **Utility**  **value** | **5Dimen-tions** | **Utility**  **value** |
| --- | --- | --- | --- | --- | --- | --- | --- | --- | --- | --- | --- | --- |
| **11111**  **11112**  **11113**  **11121**  **11122**  **11123**  **11131**  **11132**  **11133**  **11211**  **11212**  **11213**  **11221**  **11222**  **11223**  **11231**  **11232**  **11233**  **11311**  **11312**  **11313**  **11321**  **11322**  **11323**  **11331**  **11332**  **11333**  **12111**  **12112**  **12113**  **12121**  **12122**  **12123**  **12131**  **12132**  **12133**  **12211**  **12212**  **12213**  **12221**  **12222** | **1.000**  **0.786**  **0.736**  **0.768**  **0.705**  **0.656**  **0.654**  **0.592**  **0.542**  **0.804**  **0.742**  **0.692**  **0.724**  **0.661**  **0.612**  **0.610**  **0.548**  **0.498**  **0.715**  **0.652**  **0.603**  **0.635**  **0.572**  **0.522**  **0.521**  **0.458**  **0.409**  **0.795**  **0.732**  **0.682**  **0.714**  **0.652**  **0.602**  **0.601**  **0.538**  **0.488**  **0.751**  **0.688**  **0.638**  **0.670**  **0.608** | **12223**  **12231**  **12232**  **12233**  **12311**  **12312**  **12313**  **12321**  **11322**  **12323**  **12331**  **12332**  **12333**  **13111**  **13112**  **13113**  **13121**  **13122**  **13123**  **13131**  **13132**  **13133**  **13211**  **13212**  **13213**  **13221**  **13222**  **13223**  **13231**  **13232**  **13233**  **13311**  **13312**  **13313**  **13321**  **13322**  **13323**  **13331**  **13332**  **13333**  **21111** | **0.558**  **0.557**  **0.494**  **0.444**  **0.661**  **0.599**  **0.549**  **0.581**  **0.518**  **0.469**  **0.467**  **0.405**  **0.355**  **0.747**  **0.684**  **0.634**  **0.666**  **0.604**  **0.554**  **0.553**  **0.490**  **0.440**  **0.703**  **0.640**  **0.590**  **0.622**  **0.560**  **0.510**  **0.509**  **0.446**  **0.396**  **0.614**  **0.551**  **0.501**  **0.533**  **0.470**  **0.421**  **0.419**  **0.357**  **0.307**  **0.774** | **21112**  **21113**  **21121**  **21122**  **21123**  **21131**  **21132**  **21133**  **21211**  **21212**  **21213**  **21221**  **21222**  **21223**  **21231**  **21232**  **21233**  **21311**  **21312**  **21313**  **21321**  **21322**  **21323**  **21331**  **21332**  **21333**  **22111**  **22112**  **22113**  **22121**  **22122**  **22123**  **22131**  **22132**  **22133**  **22211**  **22212**  **22213**  **22221**  **22222**  **22223** | | **0.711**  **0.661**  **0.693**  **0.631**  **0.581**  **0.580**  **0.517**  **0.467**  **0.730**  **0.667**  **0.617**  **0.649**  **0.587**  **0.537**  **0.536**  **0.473**  **0.423**  **0.640**  **0.578**  **0.528**  **0.560**  **0.497**  **0.448**  **0.446**  **0.384**  **0.334**  **0.720**  **0.657**  **0.608**  **0.640**  **0.577**  **0.527**  **0.526**  **0.463**  **0.414**  **0.676**  **0.613**  **0.564**  **0.596**  **0.533**  **0.483** | **22231**  **22232**  **22233**  **22311**  **22312**  **22313**  **22321**  **22322**  **22323**  **22331**  **22332**  **22333**  **23111**  **23112**  **23113**  **23121**  **23122**  **23123**  **23131**  **23132**  **23133**  **23211**  **23212**  **23213**  **23221**  **23222**  **23223**  **23231**  **23232**  **23233**  **23311**  **23312**  **23313**  **23321**  **23322**  **23323**  **23331**  **23332**  **23333**  **31111**  **31112** | **0.482**  **0.419**  **0.370**  **0.587**  **0.524**  **0.474**  **0.506**  **0.444**  **0.394**  **0.393**  **0.330**  **0.280**  **0.672**  **0.609**  **0.560**  **0.592**  **0.529**  **0.479**  **0.478**  **0.415**  **0.366**  **0.628**  **0.565**  **0.516**  **0.548**  **0.485**  **0.435**  **0.434**  **0.371**  **0.322**  **0.539**  **0.476**  **0.426**  **0.459**  **0.396**  **0.346**  **0.345**  **0.282**  **0.232**  **0.430**  **0.367** | **31113**  **31121**  **31122**  **31123**  **31131**  **31132**  **31133**  **31211**  **31212**  **31213**  **31221**  **31222**  **31223**  **31231**  **31232**  **31233**  **31311**  **31312**  **31313**  **31321**  **31322**  **31323**  **31331**  **31332**  **31333**  **32111**  **32112**  **32113**  **32121**  **32122**  **32123**  **32131**  **32132**  **32133**  **32211**  **32212**  **32213**  **32221**  **32222**  **32223**  **32231** | **0.318**  **0.350**  **0.287**  **0.237**  **0.236**  **0.173**  **0.124**  **0.386**  **0.323**  **0.274**  **0.306**  **0.243**  **0.193**  **0,192**  **0.129**  **0.080**  **0.297**  **0.234**  **0.184**  **0.216**  **0.154**  **0.104**  **0.103**  **0.040**  **-0.010**  **0.376**  **0.314**  **0.264**  **0.296**  **0.233**  **0.184**  **0.182**  **0.120**  **0.070**  **0.332**  **0.270**  **0.220**  **0.252**  **0.189**  **0.140**  **0.138** | **32232**  **32233**  **32311**  **32312**  **32313**  **33122**  **33123**  **33131**  **32321**  **32322**  **32323**  **32331**  **32332**  **32333**  **33111**  **33112**  **33113**  **33121**  **33132**  **33133**  **33211**  **33212**  **33213**  **33221**  **33222**  **33223**  **33231**  **33232**  **33233**  **33311**  **33312**  **33313**  **33321**  **33322**  **33323**  **33331**  **33332**  **33333** | **0.076**  **0.026**  **0.243**  **0.180**  **0.131**  **0.185**  **0.136**  **0.134**  **0.163**  **0.100**  **0.050**  **0.049**  **-0.014**  **-0.063**  **0.328**  **0.266**  **0.216**  **0.248**  **0.072**  **0.022**  **0.284**  **0.222**  **0.172**  **0.204**  **0.141**  **0.092**  **0.090**  **0.028**  **-0.022**  **0.195**  **0.132**  **0.083**  **0.115**  **0.052**  **0.002**  **0.001**  **-0.062**  **-0.111** |
